# Supplementary material for: Multimodal integrated knowledge transfer to large language models through preference optimization with biomedical applications
Source: Patterns (N Y). 2026 May 4;7(8):101544. doi: 10.1016/j.patter.2026.101544 (PMC13494610; doi:10.1016/j.patter.2026.101544)
Supplement: Supplementary file 1 [file mmc1.pdf]

**Patterns, Volume *7***

## Supplemental information

# Multimodal integrated knowledge transfer to large language models through preference optimization with biomedical applications

**Zhanliang Wang, Da Wu, Quan M. Nguyen, Zhuoran Xu, and Kai Wang**

## Supplementary Materials

**Figure S1: Case studies showing how MINT improves disambiguation in rare disease prediction and tissue type classification.**

(**A) Rare disease prediction case study (text-only).** The input describes a 2-year-old female patient with Cornelia de Lange Syndrome (CdLS), including generic phenotypes such as intellectual disability and hirsutism, as well as the more specific facial feature synophrys. The SFT baseline is misled by overlap in generic symptoms and incorrectly ranks Hypertrichosis first. In contrast, MINT correctly ranks Cornelia de Lange Syndrome first, suggesting that it better prioritizes discriminative features such as synophrys to rule out hard-negative confusers.

**(B) Tissue type classification case study (vision-only).** The input image shows Colon tissue with glandular epithelial structures that are morphologically similar to Bile Duct tissue. The SFT baseline incorrectly ranks Bile Duct first, whereas MINT correctly ranks Colon first and demotes the visually similar confuser Bile Duct to rank 5. This example illustrates that preference optimization helps sharpen the decision boundary between structurally related tissue categories.

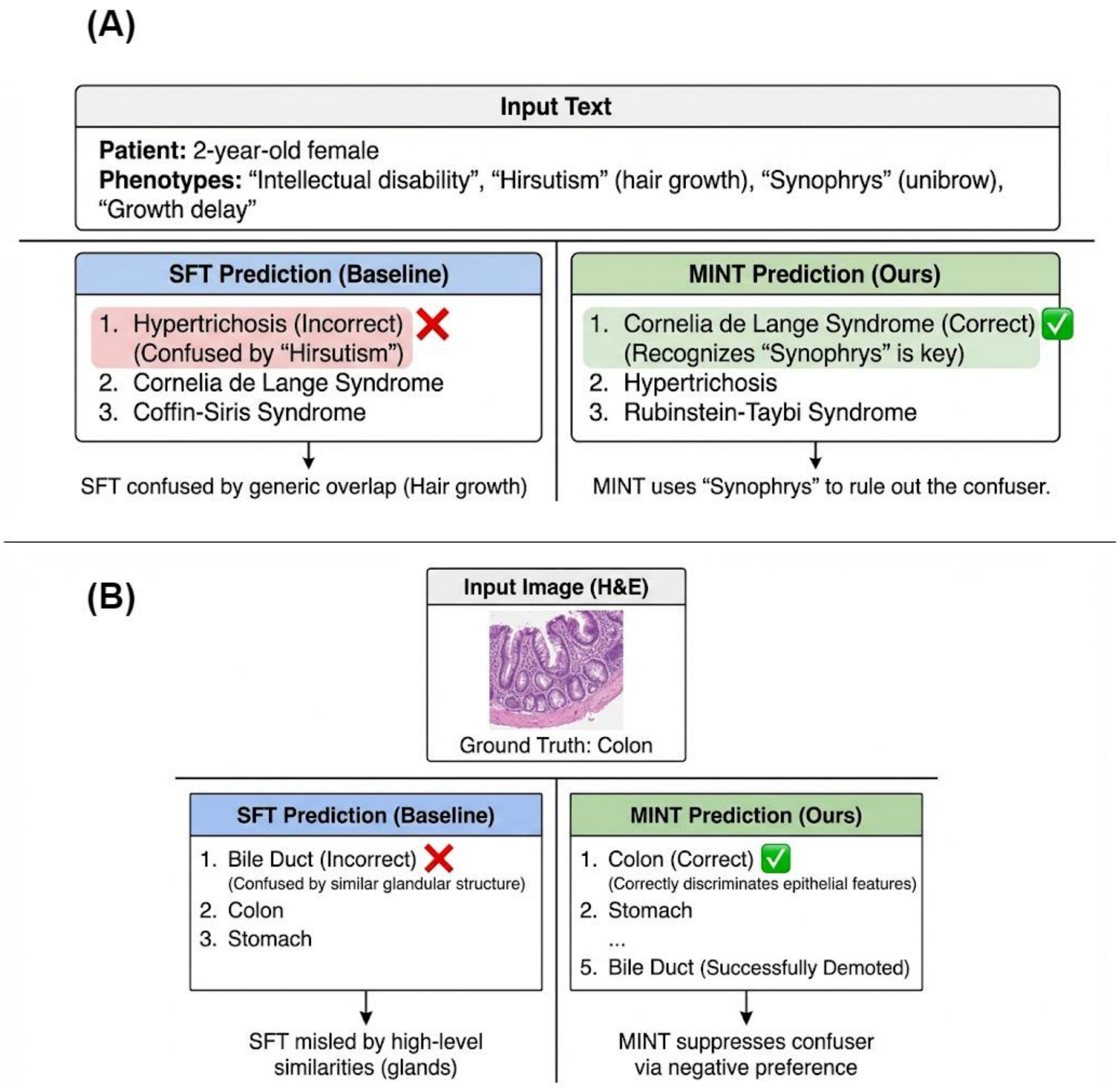

**Table S1. Main Results on Performance of various Open-Source LLMs on Rare Genetic Diseases prediction.** The mean and standard deviation are computed using three different random seeds. **'MINT' refers to our default implementation of the MINT framework using ORPO.**

| Base Model | Add-on Technique | HFA (%) | Top-10 (%) | Top-1 (%) | $CAR_{(k=10,q=10,}$ |
|---|---|---|---|---|---|
| Llama 3.2-1B-Instruct | N/A | 95.04±1.15 | 4.24±0.23 | 0.65±0.18 | 0.0292±0.0016 |
| Llama 3.2-1B-Instruct | +RAG | 76.68±4.74 | 2.21±0.17 | 0.94±0.22 | 0.0271±0.0003 |
| Llama 3.2-1B-Instruct | +SFT | 88.82±3.60 | 8.30±0.01 | 1.08±0.05 | 0.0255±0.0019 |
| Llama 3.2-1B-Instruct | +DPO | 89.22±0.37 | 7.90±0.18 | 1.63±0.26 | 0.1785±0.0054 |
| Llama 3.2-1B-Instruct | +MINT | 99.48±0.37 | 34.28±1.21 | 23.91±3.41 | 0.2120±0.0018 |
| Llama 3.2-3B-Instruct | N/A | 91.61±0.59 | 5.19±0.01 | 1.06±0.02 | 0.0522±0.0025 |
| Llama 3.2-3B-Instruct | +RAG | 98.90±0.28 | 6.52±0.05 | 0.91±0.18 | 0.0517±0.0049 |
| Llama 3.2-3B-Instruct | +SFT | 99.71±0.42 | 37.53±0.68 | 26.86±0.12 | 0.1665±0.0051 |
| Llama 3.2-3B-Instruct | +DPO | 99.63±0.21 | 38.49±0.58 | 27.07±0.19 | 0.2780±0.0016 |
| Llama 3.2-3B-Instruct | +MINT | 99.91±0.15 | 52.99±1.69 | 43.49±0.57 | 0.2877±0.0035 |
| Llama 3.1-8B-Instruct | N/A | 95.07±1.45 | 6.63±0.21 | 1.67±0.17 | 0.0797±0.0005 |
| Llama 3.1-8B-Instruct | +RAG | 99.61±0.55 | 10.95±0.10 | 1.92±0.16 | 0.0776±0.0006 |
| Llama 3.1-8B-Instruct | +SFT | 87.57±3.91 | 13.86±1.27 | 6.52±0.37 | 0.1415±0.0009 |

| Base Model | Add-on Technique | HFA (%) | Top-10 (%) | Top-1 (%) | $CAR_{(k=10,q=10,}$ |
|---|---|---|---|---|---|
| Llama 3.1-8B-Instruct | +DPO | 84.93±0.43 | 14.02±0.19 | 5.94±0.56 | 0.1618±0.0100 |
| Llama 3.1-8B-Instruct | +MINT | 97.28±2.02 | 25.91±3.29 | 16.97±0.92 | 0.2326±0.0009 |
| Gemma-2-2B-Instruct | N/A | 100.00±0.00 | 6.99±0.00 | 1.79±0.03 | 0.1065±0.0063 |
| Gemma-2-2B-Instruct | +RAG | 98.07±1.36 | 9.84±0.23 | 2.01±0.03 | 0.1100±0.0033 |
| Gemma-2-2B-Instruct | +SFT | 89.14±1.27 | 7.60±0.25 | 5.13±0.08 | 0.1259±0.0017 |
| Gemma-2-2B-Instruct | +DPO | 85.70±3.41 | 7.48±0.08 | 5.07±0.00 | 0.1737±0.0061 |
| Gemma-2-2B-Instruct | +MINT | 99.04±1.36 | 16.32±0.00 | 8.04±0.01 | 0.1743±0.0051 |
| Gemma-2-9B-Instruct | N/A | 99.56±0.63 | 12.37±0.82 | 5.03±0.16 | 0.1368±0.0047 |
| Gemma-2-9B-Instruct | +RAG | 96.71±0.01 | 10.10±0.00 | 2.99±0.00 | 0.1104±0.0001 |
| Gemma-2-9B-Instruct | +SFT | 91.98±0.01 | 14.12±0.04 | 5.29±0.02 | 0.1331±0.0011 |
| Gemma-2-9B-Instruct | +DPO | 92.07±1.68 | 13.91±0.19 | 4.88±0.07 | 0.2315±0.0023 |
| Gemma-2-9B-Instruct | +MINT | 99.61±0.55 | 33.80±1.25 | 23.40±0.85 | 0.2465±0.0122 |
| Llama 3.1-70B-Instruct | N/A | 99.74±0.16 | 21.24±0.27 | 11.92±0.08 | 0.1122±0.0031 |
| Llama 3.1-405B-Instruct | N/A | 100.00±0.00 | 26.17±0.22 | 11.66±0.08 | 0.1221±0.0019 |

| Base Model | Add-on Technique | HFA (%) | Top-10 (%) | Top-1 (%) | $CAR_{(k=10,q=10,}$ |
|---|---|---|---|---|---|
| Llama 3.3-70B-Instruct | N/A | 100.00±0.00 | 26.94±0.21 | 11.66±0.08 | 0.1221±0.0019 |

**Table S2: Performance of MINT on Llama3.2-3B-Instruct for Rare Disease Prediction Across Different Acceptance-over-Rejection (AoR) ratios $k/q$.** Recall that $k$ denotes the number of positive labels, while $q$ represents the number of negative samples within our preference-based dataset generated using GestaltMML. In this experiment, $k$ is consistently set to 10 across all ratio variations.

| k/q | HFA | Top-10 | Top-1 | $CAR_{(k=10,q=10,\lambda=1)}$ |
|---|---|---|---|---|
| 10/10 | 100.00 | 53.88 | 44.04 | 1.63 |
| 10/15 | 99.48 | 38.13 | 28.29 | 1.47 |
| 10/20 | 98.96 | 26.06 | 15.96 | 1.38 |
| 10/25 | 99.97 | 21.07 | 10.99 | 1.25 |
| 10/30 | 100.00 | 20.21 | 10.36 | 1.21 |

**Table S3. Main Results on Performance of Llama 3.2-11B-Vision-Instruct on Tissue Type Classification.** The mean and standard deviation are computed using three different random seeds. 'MINT' refers to our default implementation of the MINT framework using ORPO.

| **Method/Metric** | **HFA (%)** | **Top-5 (%)** | **Top-1 (%)** | $\boldsymbol{CAR}_{(k=5,q=5,\lambda=}$ |
|---|---|---|---|---|
| Llama 3.2-11B-Vision-Instruct | 100.00 | 32.21±0.0784 | 16.96±0.0900 | 0.2965±0.0068 |
| Llama 3.2-11B-Vision-Instruct +SFT | 100.00 | 41.46±0.0900 | 13.22±0.1700 | 0.4065±0.0037 |
| Llama 3.2-11B-Vision-Instruct +DPO | 100.00 | 41.46±0.1700 | 13.91±0.0850 | 0.5196±0.0079 |
| Llama 3.2-3B-Instruct +MINT | 100.00 | 57.58±0.195 | 28.41±0.0850 | 0.5203±0.0043 |

**Table S4. Overview of Prompts Utilized in This Study.** For SFT and MINT, which involve supervised training, labels (responses) are also included. For inference, only the prompts are presented. **'MINT' refers to our default implementation of the MINT framework using ORPO.**

| Techniques | Medical Task | Sample Prompt Content |
|---|---|---|
| **Direct Inference** | Rare Disease prediction | **System Message:** *"You are a genetic counselor. Your task is to identify potential rare diseases based on given phenotypes. Follow the output format precisely."*<br><br>**User Message:** *"A 1 year(s) 0 month(s) old female patient of Caucasian descent exhibits the following phenotypes: long face global developmental delay overgrowth, soft, doughy skin abnormality of the cardiovascular system, abnormality of the kidney scoliosis. What are the potential diagnoses?*<br><br>*Based on this information, provide a numbered list of EXACTLY 10 potential rare diseases.*<br><br>*Use EXACTLY this format:*<br><br>*POTENTIAL_DISEASES:*<br><br>*\n1. 'Disease1'\n2. 'Disease2'\n3. 'Disease3'\n4. 'Disease4'\n5. 'Disease5'\n6. 'Disease6'\n7. 'Disease7'\n8. 'Disease8'\n9. 'Disease9'\n10. 'Disease10'*<br><br>*Ensure all disease names are in single quotes, and there are exactly 10 in the list. Do not deviate from this format or add any explanations."* |
| **Direct Inference** | Tissue Type Classification | **System Message:**<br>*"You are a professional pathologist analyzing nucleus images. For each image, identify the most likely tissue types based on the nuclear morphology. Follow the output format precisely."*<br>**User Message:**<br>*Based on the morphological features observed in the provided nucleus image, which tissue types could this nucleus originate from?:*<br>*Use EXACTLY this format:*<br><br>*MOST_LIKELY_TISSUE:*<br>*\n1. 'tissue type 1'\n2. 'tissue type 2'\n3. 'tissue type 3'\n4. 'tissue type 4'\n5. 'tissue type 5'*<br><br>*Ensure all tissue types are in single quotes, and List exactly 5 tissue types in decreasing order of likelihood. Do not deviate from this format or add any explanations.* |
| **SFT** | Rare Disease prediction | **System Message:** *"You are a genetic disease diagnosis assistant."*<br><br>**User Message:** *"A 1 year(s) 0 month(s) old female patient of Caucasian descent exhibits the following phenotypes: long face global developmental delay overgrowth, soft, doughy skin abnormality of the cardiovascular system, abnormality of the kidney scoliosis. What are the potential diagnoses?"* |

| | | |
|---|---|---|
| | | **Response:** *POTENTIAL_DISEASES:\n1. hypoplastic left heart syndrome\n2. cutis laxa\n3. geroderma osteodysplasticum; go\n4. phelan-mcdermid syndrome; phmds\n5. simpson-golabi-behmel syndrome, type 1; sgbs1\n6. kbg syndrome; kbgs\n7. ogden syndrome; ogdns\n8. neurodevelopmental disorder with neonatal respiratory insufficiency, hypotonia, and feeding difficulties; nedrihf\n9. mucolipidosis ii alpha/beta\n10. williams-beuren syndrome; wbs* |
| **SFT** | Tissue Type Classification | **User Message: "***Based on the morphological features observed in the provided nucleus image, which tissue types could this nucleus originate from?"*<br>**Input Image:**<br>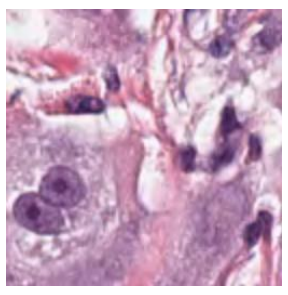<br>**Response:** '*MOST_LIKELY_TISSUE:\n\n1. Breast\n2. Colon\n3. Head & Neck\n4. Testis\n5. Prostate*' |
| **MINT** | Rare Disease prediction | **System message**: *You are a genetic disease diagnosis assistant*<br>**Prompt:**<br>*A 3 years and 6 months old male patient of European descent exhibits the following phenotypes: long palpebral fissure, growth delay, decreased body weight, eversion of lateral third of lower eyelids, feeding difficulties. What are the most likely diagnoses?*<br>**Chosen Response:**<br>*POTENTIAL_DISEASES:\n1. hypoplastic left heart syndrome\n2. cutis laxa\n3. geroderma osteodysplasticum; go\n4. phelan-mcdermid syndrome; phmds\n5. simpson-golabi-behmel syndrome, type 1; sgbs1\n6. kbg syndrome; kbgs\n7. ogden syndrome; ogdns\n8. neurodevelopmental disorder with neonatal respiratory insufficiency, hypotonia, and feeding difficulties; nedrihf\n9. mucolipidosis ii alpha/beta\n10. williams-beuren syndrome; wbs*<br>**Rejected Response**<br>*POTENTIAL_DISEASES:\n1. alzahrani-kuwahara syndrome; alkus\n2. microcephalic osteodysplastic primordial dwarfism, type ii; mopd2\n3. neurodevelopmental disorder with structural brain anomalies and dysmorphic facies; nedbaf\n4. cerebral creatine deficiency syndrome\n5. cardiac, facial, and digital anomalies with developmental delay; cafdadd\n6. clark-baraitser syndrome; clabars\n7. neurodevelopmental disorder with hypotonia and cerebellar atrophy, with or without seizures; nedhcas\n8. intellectual developmental disorder, autosomal recessive 5; mrt5\n9. robinow syndrome\n10. developmental and epileptic encephalopathy 76; dee76*' |

| | | |
|---|---|---|
| **MINT** | Tissue Type Classification | **User Message:** *"Based on the morphological features observed in the provided nucleus image, which tissue types could this nucleus originate from?"*<br>**Input Image:**<br>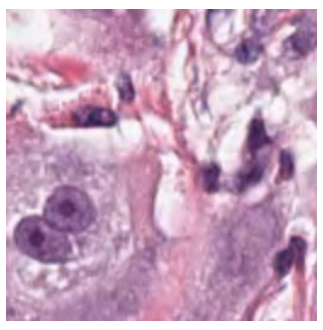<br>**Chosen Response:** '*MOST_LIKELY_TISSUE:\n\n1. Breast\n2. Colon\n3. Head & Neck\n4. Testis\n5. Prostate*'<br>**Rejected Response:** '*MOST_LIKELY_TISSUE:\n\n1. Stomach\n2. Thyroid\n3. Skin\n4. Cervix\n5. Kidney*' |

**Table S5. Disjoint disease label space analysis.** We compare the 396 unique ground-truth diseases in the Phenopacket disjoint split against the diseases appearing in the preference candidates extracted from the upstream construction; 377/396 (95.2%) never appear in either the chosen or rejected lists, and 19/396 (4.8%) appear in both (chosen-only = 0; rejected-only = 0).

| Split | #Unique GT diseases | % in chosen | % in rejected | % in both | % never |
|---|---|---|---|---|---|
| Disjoint | 396 | 4.8% (19/396)* | 4.8% (19/396)* | 4.8% (19/396) | 95.2% (377/396) |

**Table S6. Sensitivity analysis of MINT performance on rare disease prediction and tissue type classification with varying preference dataset ratios.** Mean and standard deviation were computed across three random seeds.

| Task | Ratio (%) | Num. | HFA (%) | Top-5/10 Accuracy (%) | Top-1 Accuracy (%) | $CAR_{(k=10,q=10,\lambda=1)}$ |
|---|---|---|---|---|---|---|
| Rare Disease Prediction (Llama 3.2-3B-Instruct) | 20% | 1,304 | 98.45 ± 0.52 | 19.32 ± 1.15 | 11.45 ± 0.85 | 0.0950 ± 0.0042 |
| Rare Disease Prediction (Llama 3.2-3B-Instruct) | 40% | 2,608 | 99.12 ± 0.35 | 34.60 ± 1.05 | 24.30 ± 0.92 | 0.1840 ± 0.0035 |
| Rare Disease Prediction (Llama 3.2-3B-Instruct) | 60% | 3,913 | 99.55 ± 0.20 | 43.20 ± 0.88 | 32.50 ± 0.75 | 0.2310 ± 0.0028 |
| Rare Disease Prediction (Llama 3.2-3B-Instruct) | 80% | 5,217 | 99.80 ± 0.15 | 49.75 ± 0.65 | 39.10 ± 0.45 | 0.2650 ± 0.0019 |
| **Rare Disease Prediction (Llama 3.2-3B-Instruct)** | **100%** | **6,522** | **99.91 ± 0.15** | **52.99 ± 1.69** | **43.49 ± 0.57** | **0.2877 ± 0.0035** |
| Tissue Type Classification (Llama 3.2-Vision-11B) | 20% | 1,087 | 100.00 ± 0.00 | 38.45 ± 0.55 | 19.12 ± 0.42 | 0.3350 ± 0.0051 |
| Tissue Type Classification (Llama 3.2-Vision-11B) | 40% | 2,174 | 100.00 ± 0.00 | 45.20 ± 0.62 | 22.35 ± 0.50 | 0.4120 ± 0.0045 |
| Tissue Type Classification (Llama 3.2-Vision-11B) | 60% | 3,262 | 100.00 ± 0.00 | 50.15 ± 0.45 | 25.10 ± 0.35 | 0.4680 ± 0.0032 |
| Tissue Type Classification (Llama 3.2-Vision-11B) | 80% | 4,349 | 100.00 ± 0.00 | 54.80 ± 0.30 | 27.20 ± 0.25 | 0.4950 ± 0.0028 |
| **Tissue Type Classification (Llama 3.2-Vision-11B)** | **100%** | **5,436** | **100.00 ± 0.00** | **57.58 ± 0.20** | **28.41 ± 0.09** | **0.5203 ± 0.0043** |

**Table S7. Performance Comparison with Domain-Specific Specialized Model (MedGemma-1.5-4b-it) on GMDB In-Distribution Test Set.** We compared the MINT-enhanced Llama-3.2-3B model against a specialized medical baseline of similar size, MedGemma-1.5-4b-it (4B parameters). The evaluation was performed on the in-distribution GMDB test set. While the specialized model outperforms the generalist base model, MINT enables the smaller generalist model to achieve superior diagnostic accuracy in the target domain.

| Model | Type | Parameters | HFA (%) | Top-10 Accuracy (%) | Top-1 Accuracy (%) |
|---|---|---|---|---|---|
| Llama-3.2-3B-Instruct (Base) | Generalist | 3B | 91.61 | 5.19 | 1.06 |
| MedGemma-1.5-4b-it | Specialized (Medical) | 4B | 99.20 | 32.45 | 19.80 |
| Llama-3.2-3B-Instruct + MINT | Generalist + MINT | 3B | **99.91** | **52.99** | **43.49** |
